# Synthesis of Unsymmetrically Condensed Benzo- and Thienotriazologermoles

**DOI:** 10.3390/molecules29112684

**Published:** 2024-06-05

**Authors:** Cong-Huan Wang, Yohei Adachi, Joji Ohshita

**Affiliations:** 1 Graduate School of Advanced Science and Engineering, Hiroshima University, Higashi-Hiroshima 739-8527, Japan; d210833@hiroshima-u.ac.jp (C.-H.W.); yadachi@hiroshima-u.ac.jp (Y.A.); 2 Division of Materials Model-Based Research, Digital Monozukuri (Manufacturing) Education and Research Center, Hiroshima University, Higashi-Hiroshima 739-0046, Japan

**Keywords:** triazole, germole, donor–acceptor interaction

## Abstract

Germoles and siloles unsymmetrically condensed with heteroaromatic units are attracting much interest. In this study, compounds containing a triazologermole core unit condensed with a benzene or thiophene ring were prepared. Thienotriazologermole was subjected to bromination to obtain the bromide, which underwent transformation via the palladium-catalyzed Stille coupling reaction to form triphenylamine-substituted thienotriazolegermole, with an effective extension of conjugation. The electronic states and properties of these triazologermole derivatives are discussed on the basis of optical and electrochemical measurements and density functional theory calculations. Triphenylamine-substituted thienotriazolegermole showed clear solvatochromic properties in photoluminescence measurements, suggesting that intramolecular charge transfer occurs at the photo-excited state. This clearly indicates that the triazologermole unit is useful as an acceptor of donor–acceptor compounds. The potential application of triphenylamine-substituted thienotriazolegermole as a sensing material was also explored.

## 1. Introduction

Triazoles are five-membered aromatic ring systems containing two double bonds and three sequentially arranged nitrogen atoms. In addition to their distinctive structural properties such as a high dipole moment, molecular rigidity, and promising photochemical characteristics, their excellent accessibility and ease of modification make triazoles highly appealing. Moreover, triazoles serve as Lewis bases to facilitate intermolecular interactions with Lewis acids. Triazole derivatives constitute an important class of heterocycles for applications in pharmaceuticals [1], biologically active agents that can interact with biologically active substances through such interactions as hydrogen bonding [2], and other functional materials such as mechanochromic, photoluminescent, and fluorosensing materials [3,4,5,6,7,8], and have been demonstrated to serve as building blocks for the synthesis of polymers, dendrimers, and other functional materials [9]. Examples include triazole-containing organic electronic materials that can be used for solar cells [10,11,12,13], field-effect transistors [14,15,16], and light-emitting diodes [17,18,19]. Materials incorporating triazole units have been developed for gas storage [20], gas separation [21], and optical brightening [22], and the potential utilization of these materials in sensors [23] and catalysts [24] has been explored. Triazole units are regarded as promising electron-acceptor candidates for the construction of donor–acceptor (D–A) compounds.

Si/Ge-condensed compounds, such as siloles and germoles, have received considerable attention in recent decades for their potential applications in organic electronics [25,26]. These structures exhibit a notable phenomenon known as σ*–π* conjugation, which is frequently observed between Si/Ge σ*-orbitals and endocyclic butadiene π*-orbitals. This interaction serves to stabilize the lowest unoccupied molecular orbital (LUMO), resulting in an enhanced electron affinity and reduced energy gaps between the highest occupied molecular orbital (HOMO) and LUMO [27,28,29]. The high planarity of siloles and germoles is also advantageous for their use as building units of conjugated materials. These characteristics have significant implications on the electronic properties of siloles and germoles. Researchers have explored strategies to fine-tune the functionalities of these compounds to allow for tailored applications in various electronic devices via condensation with aromatic ring systems [30]. Currently, dithienosilole and dithienogermole are used as building units for preparing conjugated functional materials for optoelectronic devices, such as OLEDs [31], organic solar cells [32], and organic transistors [33]. However, most condensed siloles and germoles reported so far possess symmetrical tricyclic systems [34], and studies on unsymmetrical tricyclic systems have been limited to simple cases, such as combinations of benzene–pyridine [35,36], benzene–thiophene [37,38], and thiophene–pyridine ring systems [26]. 

In this work, we designed and synthesized two types of germoles unsymmetrically condensed with benzene–triazole and thiophene–triazole units (**BTAG** and **TTAG**), with the hope that D–A interactions between benzene/thiophene and triazole units linked by a germole system would allow for the development of building units of conjugated functional materials having finely tuned electronic states. The optical and electrochemical properties of these triazologermole derivatives were systematically investigated. We also prepared **TTAG** bromide as a precursor of various **TTAG**-containing conjugated materials. Indeed, **TTAG** bromide underwent the palladium-catalyzed Stille coupling reaction to furnish triphenylamine-substituted **TTAG** (**TTAG-TPA**) with the effective extension of conjugation. The electronic states of **TTAG-TPA** were investigated by optical and electrochemical measurements and density functional theory (DFT) calculations. Although **BTAG** and **TTAG** showed no evident intramolecular D–A interaction, **TTAG-TPA** exhibited solvatochromic properties attributable to the intramolecular D–A interaction between the triphenylamine and triazole units, with a shift of the photoluminescence (PL) band toward lower energies with increasing solvent polarity. These results suggest potential applications of triazologermole units as building units of conjugated materials. 

## 2. Results and Discussion

### 2.1. Synthesis

**Scheme 1:**
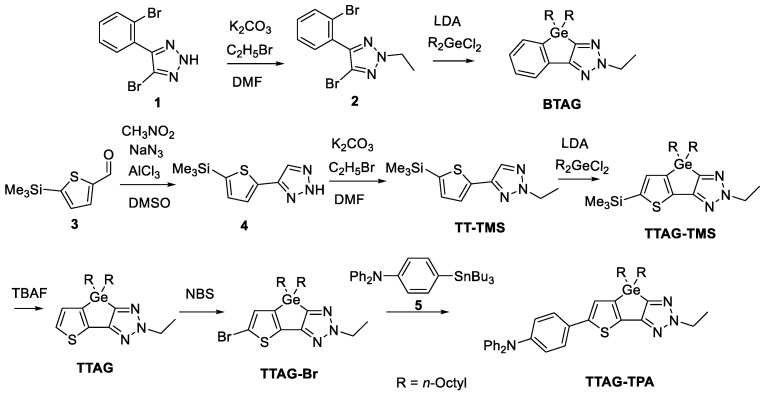
Synthesis of **BTAG**, **TTAG**, **TTAG-TMS**, and **TTAG-TPA**.

The synthetic strategy is shown in Scheme 1. Compound **1** was prepared following a previously reported method [39] and used for the subsequent synthesis of **BTAG** via compound **2**. **TT-TMS** was obtained by the ethyl substitution of **4** prepared from **3**. Compound **3** was prepared as previously reported [40]. The dilithiation of **TT-TMS** using LDA, followed by a ring-forming reaction with dichlorodi(*n*-octyl)germane, resulted in a mixture containing **TTAG-TMS**, unsubstituted **TT-TMS**, and numerous unidentified products. From the mixture, **TTAG-TMS** was isolated in 58% yield by silica gel column chromatography. **TTAG-TMS** was treated with TBAF to obtain **TTAG** with a yield of 85%. The bromination of **TTAG** with NBS gave **TTAG-Br**, which was further subjected to palladium-catalyzed Stille coupling with **5** to obtain **TTAG-TPA**.

### 2.2. Optical and Electrochemical Properties

**Figure 1:**
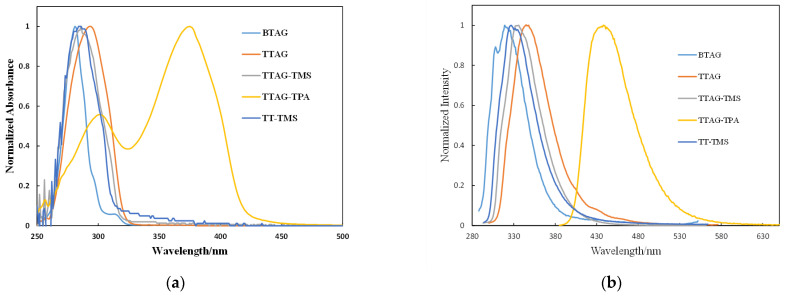
UV–vis absorption spectra (**a**) and PL spectra (**b**) of triazologermoles in THF (1.0 × 10^−5^ M) at room temperature.

**Figure 2:**
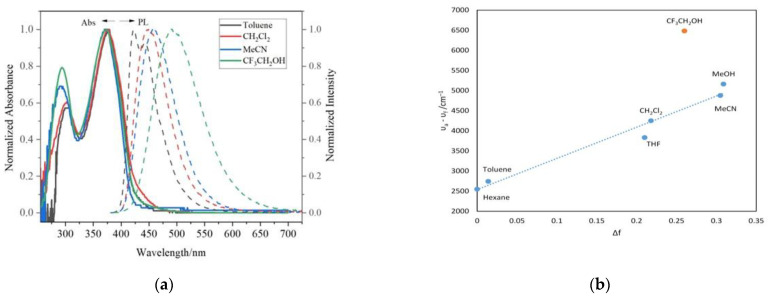
UV–vis absorption (solid line) and solvatochromic PL (dotted line) study (**a**) and the Lippert–Mataga plot (**b**) of **TTAG-TPA**. The slope of the fitted line is 7747 cm^−1^ (R^2^ = 0.97).

We examined the optical and electrochemical properties of the prepared triazologermole derivatives to clarify their electronic states. The UV–vis absorption and PL spectra of **BTAG**, **TTAG**, **TTAG-TMS**, and **TTAG-TPA** in THF are shown in Figure 1, and the corresponding photophysical data are summarized in Table 1. **BTAG** displayed a maximum absorption at 281 nm in THF with a shoulder at 290 nm. Replacing benzene with thiophene in **TTAG** caused a bathochromic shift of 13 nm to give an absorption maximum at 294 nm in THF. When compared with thienopyridinogermole, the absorption maximum of **TTAG** was shifted to the shorter wavelength region by 6 nm, reflecting the less extended conjugation of thienyltriazole than thienylpyridine [26]. In fact, 5-trimethylsilyl-2-thienylpyridine possesses the absorption maximum at 324 nm, redshifted by approximately 40 nm compared to that of **TT-TMS** [26]. Silyl substitution at the α-position of the thiophene ring in **TTAG-TMS** shifted the absorption maximum slightly to 286 nm. This finding differed from a previous report on DTS derivatives that found a shift of absorption bands to lower energies because of a similar silyl substitution [41]. Germole condensation in **TTAG-TMS** resulted in a red shift of the PL band by 9 nm relative to that of **TT-TMS**. However, despite our expectation, their UV absorption bands were at nearly the same energies. Compounds **BTAG**, **TTAG**, and **TTAG-TMS** showed weak PL. Figure S1a–c shows the absorption spectra of **BTAG**, **TTAG**, and **TTAG-TMS** recorded in different solvents. Only negligible solvatochromic shifts were observed in both the UV–vis and PL spectra of these compounds, indicating the absence of evident D–A interactions. **TTAG-TPA** exhibited an absorption band and a PL band at 375 nm and 438 nm in THF, respectively, both of which were largely red-shifted compared with those of **BTAG**, **TTAG**, and **TTAG-TMS**. It is likely that the introduction of the triarylamine substituent on the thiophene ring efficiently enhanced conjugation. In the UV–vis absorption spectrum, a band at 300 nm was also observed in THF, which is possibly due to the local excitation regarding the **TTAG** unit. The PL bands have intramolecular charge transfer (ICT) characteristics, which can be verified by the solvatochromic effects, as shown in Figure 2. For example, the PL band of **TTAG-TPA** was observed at 423 nm in toluene, which was bathochromically shifted to 456 nm in more polar MeCN. In contrast, the PL spectra of **BTAG**, **TTAG**, and **TTAG-TMS** showed no clear solvatochromism. The red shift of the PL band with increasing solvent polarity clearly indicates that the excited states were more polar than the ground state [42]. In fact, nearly no evident solvatochromic effects were observed in the UV–vis absorption spectra of **TTAG-TPA**. To further investigate the ICT characteristics of **TTAG-TPA**, the Stokes shift (*ν*_a_ − *ν*_f_) in each solvent was plotted as a function of its orientational polarizability Δ*f* to obtain Lippert–Mataga plots [43,44]. A linear relationship between the Stokes shifts and orientational polarizabilities was observed (Figure 2 and Table S1). Using the slope of the Lippert–Mataga plot, the dipole moment change (Δ*μ*) upon photoexcitation was calculated to be 18.4 D (Figure S2) [26]. This was smaller than that calculated for thienopyridinogermole with the same substituents (19.9 D), suggesting that fine tuning of the D–A interaction is possible by changing the acceptor unit.

**TTAG-TPA** had a higher PL quantum yield than the other triazologermole derivatives, likely reflecting the extended conjugation. **TTAG-TPA** exhibited blue PL in all solvents with maxima in the range of 410–490 nm, except in trifluoroethanol in which green emission was observed (Figure S3). Interestingly, the Stokes shift of **TTAG-TPA** in trifluoroethanol deviated from that predicted from the Lippert–Mataga plot of **TTAG-TPA** in different solvents, and the largely red-shifted PL band at 492 nm suggested a specific interaction of **TTAG-TPA** with trifluoroethanol. For instance, hydrogen bonding might have resulted in the complex formation of **TTAG-TPA** with trifluoroethanol, as previously reported for triphenylamine-substituted thienopyridinogermole [26].

Anodic cyclic voltammograms (CVs) were examined to investigate the redox characteristics and HOMO energy levels of triazologermole derivatives (Table 1 and Figure S4). The oxidation peak of **TTAG-TMS** was found at 1.62 V (vs. Fc^+^/Fc), lower than that of **TT-TMS** (1.84 eV), indicating that the introduction of the germole unit raised the HOMO energy level. The oxidation peak of **BTAG** was observed at 1.96 V, higher than that of **TTAG** (1.57 V), further supporting the less extended conjugation in **BTAG**. The first oxidation peak (E0ox1) of **TTAG-TPA** was much lower than that of **TTAG**, at 1.04 V. The HOMO energy levels determined from the onsets of the first oxidation were −6.12 eV for **BTAG**, −5.80 eV for **TTAG**, −5.50 eV for **TTAG-TMS**, and −5.23 for **TTAG-TPA**. It should be noted that the introduction of the triphenylamine unit had a significant effect on the elevation of the HOMO energy level. This can be attributed to the contribution of the strong electron-donating triphenylamine group. In contrast, silyl substitution at the α-position of the thiophene ring in **TTAG-TMS** lowered the HOMO energy level.

### 2.3. DFT Calculations

**Figure 3:**
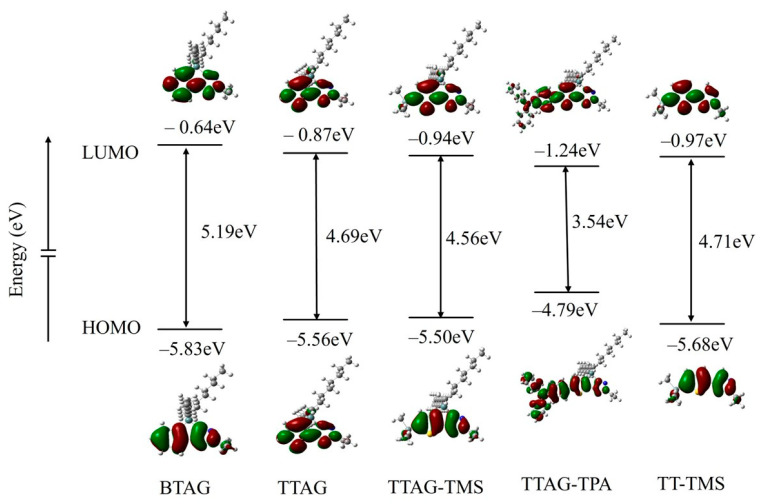
Energy levels and profiles of **BTAG**, **TTAG**, **TTAG-TMS**, **TTAG-TPA**, and **TT-TMS** derived from DFT calculations at the B3LYP/6-31G(d,p) level of theory in the gas phase.

To clarify the electronic states of triazologermole derivatives, we carried out DFT calculations using the Gaussian 16 suite of quantum chemical simulation programs for condensed germoles. First, structures were optimized in vacuo using DFT calculations at the B3LYP/6-31G(d,p) level [45]. Figure 3 shows the results of DFT calculations. The HOMO of **BTAG** is based on π-conjugation. The higher HOMO of **TTAG** than that of **BTAG** by 0.27 eV is likely due to electron donation by the thiophene unit of **TTAG**, which suggests the higher Lewis basicity of **TTAG** than **BTAG**. At the same time, the LUMO is lowered by 0.23 eV relative to that of **BTAG**. The LUMOs of triazologermole derivatives show σ*–π* interaction, particularly between the thiophene β-carbon and the germanium atom. However, orbital overlapping is limited despite our expectations, with the LUMO of **TTAG-TMS** being at a higher energy than that of **TT-TMS**. The reduced HOMO–LUMO energy gap of **TTAG-TMS** compared with that of **TT-TMS** appears to be primarily due to the electron-donating properties of the germanium unit, raising the HOMO energy level. 

The HOMO–LUMO energy gaps decreased in the order of **BTAG** > **TT-TMS** > **TTAG** > **TTAG-TMS** > **TTAG-TPA**, in accordance with their optical properties, except for **TTAG-TMS** (**TTAG-TMS** showed a smaller optical band gap than **TTAG**). The CVs showed a higher anodic potential for **TTAG-TMS** than for **TTAG**, indicating a shallower HOMO energy level. These findings are inconsistent with the results of computation, suggesting the contribution of trimethylsilyl substitution to both the HOMO and LUMO to decrease the energy gap, although the degree of contribution seemed small. Unfortunately, we have no direct data to explain this discrepancy between the experimental and computational data for **TTAG-TMS**. The introduction of the triphenylamine group to the **TTAG** unit affected the HOMO−LUMO energy gap in **TTAG-TPA** mainly by raising the HOMO energy level, rather than lowering the LUMO energy level. Indeed, the LUMO of **TTAG-TPA** was lower only by 0.37 eV than that of **TTAG**, while the HOMO was higher by 0.77 eV. In **TTAG-TPA**, the HOMO is mainly on the (diphenylaminophenyl)thiophene unit, whereas the LUMO is distributed more on the triazologermole unit, suggesting that charge separation may occur in the photoexcited state. However, the separated distribution of the HOMO and LUMO was not observed in **BTAG**, **TTAG**, and **TTAG-TMS**. This finding is in line with the fact that the UV absorption and PL bands of these compounds exhibited no evident solvatochromic effects. 

## 3. Experimental Section

### 3.1. General Preparations

All reactions were carried out under a dry argon atmosphere. *N*,*N*-dimethylformamide (DMF) and tetrahydrofuran (THF) were distilled from CaH_2_ and stored over activated molecular sieves in the dark until use as reaction solvents. Synthetic procedures for compounds **2** and **4** are presented in the Supporting Information. Compound **5** was prepared as reported in study [46]. Nuclear magnetic resonance (NMR) spectra were recorded on a Varian 400-MR spectrometer (Palo Alto, CA, USA). NMR spectra of newly prepared compounds are presented in the Supporting Information. UV–vis absorption and PL spectra in various solvents were recorded at room temperature using Hitachi U-2910 (Tokyo, Japan) and HORIBA FluoroMax-4 spectrophotometers (Kyoto, Japan), respectively. APCI-mass spectra were obtained with a Thermo Fisher Scientific LTQ Orbitrap XL spectrometer (Waltham, MA, USA) at N-BARD, Hiroshima University. Electrochemical redox potentials were obtained by cyclic voltammetry measurements performed with a computer-controlled Autolab analyzer (Kyoto, Japan) using a typical three-electrode electrochemical cell in a solution of tetrabutylammonium hexafluorophosphate (0.1 M) in anhydrous acetonitrile at a scan rate of 50 mV s^−1^ at room temperature under nitrogen. A Pt disk was used as the working electrode, a Pt wire as the counter electrode, and an Ag/Ag^+^ electrode as the reference electrode. The potential of the reference electrode was calibrated with ferrocene.

### 3.2. Synthetic Procedures

#### 3.2.1. Synthesis of **BTAG**

To a solution of **2** (0.507 g, 1.532 mmol) in 20 mL of THF, 1.915 mL of *n*-BuLi (1.6 M) in hexane at −78 °C was added, and the mixture was stirred at this temperature for 2 h. Dichlorodi(*n*-octyl)germane (0.623 g, 1.685 mmol) was added to the mixture at −78 °C. After stirring overnight at room temperature, the solvent was evaporated under reduced pressure, and the residue was directly purified by column chromatography on silica gel with EtOAc/hexane (1:10) as the eluent to give **BTAG** as a colorless oil in 65% yield (0.490 g, 1.042 mmol). ^1^H NMR (400 MHz, CDCl_3_) δ 7.79 (d, *J* = 7.5 Hz, 1H), 7.53 (d, *J* = 7.1 Hz, 1H), 7.40 (t, *J* = 7.5 Hz, 1H), 7.28–7.25 (m, 1H), 4.56 (q, *J* = 7.3 Hz, 2H), 1.62 (t, *J* = 7.3 Hz, 3H), 1.47–1.39 (m, 4H), 1.30–1.16 (m, 24H), 0.85 (t, *J* = 7.0 Hz, 6H). ^13^C NMR (100 MHz, CDCl_3_) δ 160.9, 148.6, 143.7, 138.1, 133.9, 129.6, 127.8, 121.9, 50.1, 32.9, 32.0, 29.3, 29.2, 25.4, 22.8, 15.4, 14.3, 14.2. HR-MS (APCI) Calcd for C_26_H_44_GeN_3_: [M + H]^+^: 472.27415, Found: 472.27499.

#### 3.2.2. Synthesis of **TT-TMS**

A mixture of **4** (0.510 g, 2.283 mmol), K_2_CO_3_ (0.316 g, 2.283 mmol), and C_2_H_5_Br (0.373 g, 3.425 mmol) was stirred in 15 mL of DMF at room temperature in air for 5 h. The solvent was evaporated in vacuo, and the residue was chromatographed on silica gel eluted with EtOAc/hexane (1:5) to give **TT-TMS** as a yellow oil in 78% yield (0.69 g, 1.41 mmol). ^1^H NMR (400 MHz, CDCl_3_) δ 7.72 (s, 1H), 7.40 (d, *J* = 3.4 Hz, 1H), 7.20 (d, *J* = 3.4 Hz, 1H), 4.49 (q, *J* = 7.3 Hz, 2H), 1.56 (t, *J* = 7.2 Hz, 3H), 0.34 (s, 9H). ^13^C NMR (100 MHz, CDCl_3_) δ 142.8, 140.9, 134.7, 130.6, 125.8, 112.2, 50.2, 15.0, 0.0. HR-MS (APCI) Calcd for C_11_H_18_N_3_SSi: [M + H]^+^: 252.09852, Found: 252.09856.

#### 3.2.3. Synthesis of **TTAG-TMS**

**TTAG-TMS** was synthesized in a similar fashion to that described for **BTAG** using compound **TT-TMS** (0.39 g, 1.551 mmol) and lithium diisopropylamide (LDA) (3.179 mmol) instead of **2** and *n*-BuLi, respectively. **TTAG-TMS** was obtained as a yellow oil in 58% yield (0.494 g, 0.899 mmol). ^1^H NMR (400 MHz, CDCl_3_) δ 6.98 (s, 1H), 4.50 (q, *J* = 7.3 Hz, 2H), 1.56 (t, *J* = 7.3 Hz, 3H), 1.45–1.38 (m, 4H), 1.37–1.13 (m, 24H), 0.85 (t, *J* = 7.0 Hz, 6H), 0.28 (s, 9H). ^13^C NMR (100 MHz, CDCl_3_) δ 148.19, 141.16, 140.68, 138.68, 134.11, 127.23, 49.91, 33.00, 32.04, 29.30, 29.16, 24.87, 22.80, 15.31, 14.26, 13.69, 0.01. HR-MS (APCI) Calcd for C_27_H_50_N_3_GeSSi: [M + H]^+^: 550.27010, Found: 550.27045.

#### 3.2.4. Synthesis of **TTAG**

To a solution of **TTAG-TMS** (0.287 g, 0.522 mmol) in THF (15 mL), a solution of tetra(*n*-butylammonium) fluoride (TBAF) (0.522 mmol, 1 M) in THF at 0 °C was added, and the mixture was stirred for 10 min at this temperature. After the evaporation of the solvent, the residue was diluted with 10 mL of ether acetate and hydrolyzed with water. The organic layer was separated, washed with 3 × 10 mL of water, and dried over anhydrous magnesium sulfate. After the evaporation of the solvent, the residue was subjected to silica gel column chromatography with hexane/CH_2_Cl_2_ (10:1) as the eluent to give TTAG as a colorless oil in 86% yield (0.214 g, 0.449 mmol). ^1^H NMR (400 MHz, CDCl_3_) δ 7.36 (d, *J* = 4.8 Hz, 1H), 7.11 (d, *J* = 4.7 Hz, 1H), 4.51 (q, *J* = 7.3 Hz, 2H), 1.59 (t, *J* = 7.2 Hz, 3H), 1.50–1.38 (m, 4H), 1.29–1.17 (m, 24H), 0.86 (t, *J* = 7.0 Hz, 6H). ^13^C NMR (100 MHz, CDCl_3_) δ 156.0, 150.6, 144.8, 140.9, 129.8, 126.9, 77.5, 77.2, 76.8, 49.9, 32.8, 32.0, 29.3, 29.2, 25.4, 22.8, 15.3, 14.5, 14.2. HR-MS (APCI) Calcd for C_24_H_42_N_3_GeS: [M + H]^+^: 478.23057, Found: 478.23077.

#### 3.2.5. Synthesis of **TTAG-Br**

A mixture of **TTAG** (0.182 g, 0.383 mmol) and *N*-bromosuccinimide (NBS) (0.068 g, 0.383 mmol) was stirred in 15 mL of CH_2_Cl_2_ at room temperature in the dark. After 10 h, the reaction was quenched with water, and the resulting mixture was extracted with CH_2_Cl_2_. The extracts were combined and washed with brine (2 × 10 mL). After drying the organic phase over anhydrous sodium sulfate, the solvent was removed under reduced pressure, and the residue was purified by column chromatography with hexane/CH_2_Cl_2_ (10:1) as the eluent to give **3** as a colorless oil in 95% yield (0.202 g, 0.363 mmol). ^1^H NMR (400 MHz, CDCl_3_) δ 7.06 (s, 1H), 4.50 (d, *J* = 7.3 Hz, 2H), 1.59 (t, *J* = 7.3 Hz, 3H), 1.47–1.38 (m, 4H), 1.30–1.17 (m, 28H), 0.86 (t, *J* = 7.0 Hz, 3H). ^13^C NMR (100 MHz, CDCl_3_) δ: 155.8, 149.5, 145.1, 141.7, 132.5, 113.3, 50.0, 32.7, 31.9, 29.3, 29.2, 25.4, 22.8, 15.3, 14.5, 14.2. HR-MS (APCI) Calcd for C_24_H_41_N_3_BrGeS: [M + H]^+^: 556.14109, Found: 556.14136.

#### 3.2.6. Synthesis of **TTAG-TPA**

Compounds **TTAG-Br** (0.13 g, 0.234 mmol) and **5** (0.084 g, 0.156 mmol) were dissolved in toluene (3 mL) that had been degassed by argon bubbling for approximately 4 h. Pd(PPh_3_)_4_ (5.41 mg, 4.68 µmol) was added and the solution was heated to reflux with stirring for 10 h. After removal of the solvent under reduced pressure, the residue was purified by flash column chromatography (SiO_2_) with hexane/CH_2_Cl_2_ (10:1) as the eluent to afford product **TTAG-TPA** as an orange oil in 40% yield (0.045 g, 0.062 mmol). ^1^H NMR (400 MHz, CDCl_3_) δ 7.49 (d, *J* = 8.8 Hz, 1H), 7.29–7.24 (m, 4H), 7.23 (s, 1H), 7.13–7.11 (m, 4H), 7.08–7.02 (m, 4H), 4.51 (q, *J* = 7.3 Hz, 2H), 1.59 (t, *J* = 7.2 Hz, 3H), 1.50–1.38 (m, 4H), 1.29–1.17 (m, 24H), 0.86 (t, *J* = 7.0 Hz, 6H). ^13^C NMR (100 MHz, CDCl_3_) δ. 156.2, 150.2, 147.6, 147.4, 146.7, 146.2, 139.2, 129.4, 128.7, 126.7, 125.0, 124.6, 123.9, 123.2, 49.9, 32.8, 32.0, 29.3, 29.2, 25.4, 22.8, 15.3, 14.5, 14.2. HR-MS (APCI) Calcd for C_42_H_55_N_3_GeS: [M + H]^+^: 721.33537, Found: 721.33685.

### 3.3. Computational Details

DFT calculations were performed using the Gaussian 16 program (Gaussian Inc., Wallingford, CT, USA). Geometrical optimization was carried out in vacuo using the B3LYP functional and the 6-31G(d,p) basis set for all atoms.

## 4. Conclusions

In conclusion, we have synthesized triazologermole compounds for the first time and thoroughly investigated their optical and electrochemical behaviors to elucidate the electronic states. Our findings suggest that replacing the condensed benzene ring of **BTAG** with a thiophene ring enhances the conjugation, as evidenced by the red-shifted absorption of **TTAG** relative to that of **BTAG**. Contrary to our expectation, germole condensation raised both HOMO and LUMO energy levels, suggesting that the σ*–π* conjugation does not play an important role in the triazologermole system and that the germole unit serves as an electron donor, unlike conventional germole compounds such as dithienogermoles and dibenzogermoles. We also found that bromo-substituted **TTAG** can be used as a potential precursor of **TTAG**-based conjugated compounds, and an electron-donating triphenylamine unit was introduced to the **TTAG** thiophene ring. The resulting **TTAG-TPA** showed the expected intramolecular D–A interaction, indicating that the **TTAG** unit can be used as an electron-accepting unit in conjugated D–A systems. Furthermore, **TTAG-TPA** had sufficient Lewis basicity for complex formation with trifluoroethanol, inducing a clear red shift of the PL band, suggesting potential applications of triazologermole derivatives as sensor materials. The triazologermole seems to be useful as an acceptor unit being applicable to donor–acceptor compounds. Further studies to explore the applications of triazologermole-based materials are underway.

## Figures and Tables

**Table 1:** Optical and electrochemical properties of triazologermoles.

Compound	UV abs. λ_max_^a^/nm	PL λ_max_^a,b^/nm (Φ_f_)	HOMO ^c^/eV	LUMO ^d^/eV	LUMO–HOMO/eV
**BTAG**	281	319 (nd ^e^)	−6.12	−1.93	4.19
**TTAG**	294	348 (nd ^e^)	−5.80	−1.91	3.86
**TTAG-TMS**	286	336 (nd ^e^)	−5.84	−1.93	3.91
**TTAG-TPA**	300, 375	438 (38%)	−5.23	−2.26	2.97
**TT-TMS**	285	327 (nd ^e^)	−6.04	−2.07	3.97

^a^ In THF at room temperature. ^b^ Excited at the absorption maximum. ^c^ Derived from CV anodic onset potential. ^d^ E (LUMO) = E (HOMO) + Eg. ^e^ Too low to determine.

## Data Availability

Data are contained within the article and Supplementary Materials.

## Supplementary Materials

The following supporting information can be downloaded at: https://www.mdpi.com/article/10.3390/molecules29112684/s1, Experimental details for the preparation of compounds **2** and **4**; Figure S1: UV absorption spectra (left) and PL spectra (right) of **BTAG** (a), **TTAG** (b), **TTAG-TMS** (c), and **TTAG-TPA** (d) in several solvents; Figure S2: The Lippert–Mataga plot (left) and structure (right) of thienopyridinogermole; Figure S3: Photo of **TTAG-TPA** in several solvents under irradiation at 365 nm at room temperature; Table S1: Absorption maxima, emission maxima and Stokes’ shift of **TTAG-TPA** in various solvents; Figure S4: Cyclic voltammograms of **BTAG**, **TTAG-TMS**, and **TTAG-TPA** in MeCN/TBAHFP (0.1 M), [c] = 1 × 10^−4^ mol L^−1^, 298K, scan rate = 50 mV s^−1^; Figures S5–S20: NMR spectra of prepared compounds in the present study. References [39,40,47,48] are cited in the Supplementary Materials.
