# Supplementary material for: Synthesis of Unsymmetrically Condensed Benzo- and Thienotriazologermoles
Source: Molecules. 2024 Jun 5;29(11):2684. doi: 10.3390/molecules29112684 (PMC11173466; doi:10.3390/molecules29112684)
Supplement: Supplementary file 1 [file molecules-29-02684-s001.zip › molecules-3041457-supplementary.pdf]

Supporting Information

Synthesis of Unsymmetrically Condensed Benzo- and Thienotriazologermoles

Cong-Huan Wang 1, Yohei Adachi 1 and Joji Ohshita 1,2,*

1 Graduate School of Advanced Science and Engineering, Hiroshima University, Higashi-Hiroshima 739-8527, Japan

2 Division of Materials Model-Based Research, Digital Monozukuri (Manufacturing) Education and Research Center, Hiroshima University, Higashi-Hiroshima 739-0046, Japan

Content

1. Experimental details for the preparation of compounds **2** and **4**. →S2
2. **Figure S1.** UV absorption (left) and PL spectra (right) of **BTAG** (a), **TTAG** (b), **TTAG-TMS** (c), **TTAG-TPA** (d) in several solvents. →S3
3. **Figure S2.** Lippert-Mataga plots (left) and the structure (right) of thienopyridinogermole. →S4
4. **Figure S3.** Photo of **TTAG-TPA** in several solvents under irradiation at 365 nm at room temperature. →S4
5. **Table S1.** Absorption maxima, emission maxima and Stokes' shift of TTAG-TPA in various solvents. →S4
6. **Figure S4.** Cyclic voltammograms of **BTAG**, **TTAG-TMS**, and **TTAG-TPA** in MeCN/TBAHFP (0.1 M), [c] = 1 × 10^{-4} mol L^{-1}, 298K, scan rate = 50 mV s^{-1}. →S5
7. **Figures S5-S20.**NMR spectra of newly prepared compounds in the present study. →S6-13

EXPERIMENTAL

General

All reactions were carried out under dry argon atmosphere. *N*, *N*-dimethylformamide (DMF) and dimethyl sulfoxide (DMSO) that were used as the reaction solvents were distilled from CaH_2 and stored over activated molecular sieves in the dark until use. Compounds **1** and **3** were prepared as reported in the literature [1-2]. Nuclear magnetic resonance (NMR) spectra were recorded on a Varian 400-MR spectrometer. APCI-mass spectra were obtained by a Thermo Fisher Scientific LTQ Orbitrap XL spectrometer at N-BARD, Hiroshima University.

*Synthesis of compound **2**.*

The mixture of **1** (0.303 g, 1.00 mmol), K_2CO_3 (0.138 g, 1.00 mmol), and C_2H_5Br (0.164 g, 1.50 mmol) was stirred in 15 mL of DMF at room temperature in air for 5 h. The solvent was evaporated in vacuo and the residue was chromatographed on silica gel with EtOAc/hexane (1:5) as the eluent to give **2** in 87% yield (0.287 g, 0.87 mmol) as a white solid. ^{1}H NMR (400 MHz, $CDCl_3$) δ: 7.70 (d, J = 8.0 Hz, 1H), 7.42-7.37 (m, 2H), 7.34-7.28 (m, 1H), 4.51 (q, J = 7.3 Hz, 2H), 1.62 (t, J = 7.3 Hz, 3H). ^{13}C NMR (100 MHz, $CDCl_3$) δ: 146.2, 133.3, 132.2, 130.9, 130.5, 127.4, 124.0, 121.9, 51.2, 14.9. HR-MS (APCI) Calcd for $C_{10}H_{10}Br_2N_3$: $[M+H]^+$: 329.92360, Found: 329.92419. The NMR spectra are presented in Figures S4 and S5.

*Synthesis of compound **4**.*

A mixture of **3** (1.20 g, 6.510 mmol), nitromethane (0.596 g, 9.765 mmol), NaN_3 (0.931 g, 14.322 mmol), and $AlCl_3$ (0.087 g, 0.651 mmol), was stirred in 20 mL DMSO at 80 ^{o}C in air overnight. The solution hydrolyzed with brine and extracted with EtOAc. The combined organic layers were dried over anhydrous sodium sulfate, and the solvent was evaporated in vacuo. The residue was chromatographed on silica gel with EtOAc/hexane (1:2) as the eluent to give **4** in 70 % yield (1.017 g, 4.557 mmol) as a brown oil. ^{1}H NMR ($CDCl_3$) δ: 7.88 (s, 1H), 7.48 (d, J = 3.4 Hz, 1H), 7.23 (d, J = 3.4 Hz, 1H), 0.35 (s, 9H). ^{13}C NMR (100 MHz, $CDCl_3$) δ 141.6, 137.0, 134.8, 129.5, 126.5, 112.2, 0.0. HR-MS (APCI) Calcd for $C_9H_{14}N_3SSi$: $[M+H]^+$: 224.06722, Found: 224.06767. The NMR spectra are presented in Figures S8 and S9.

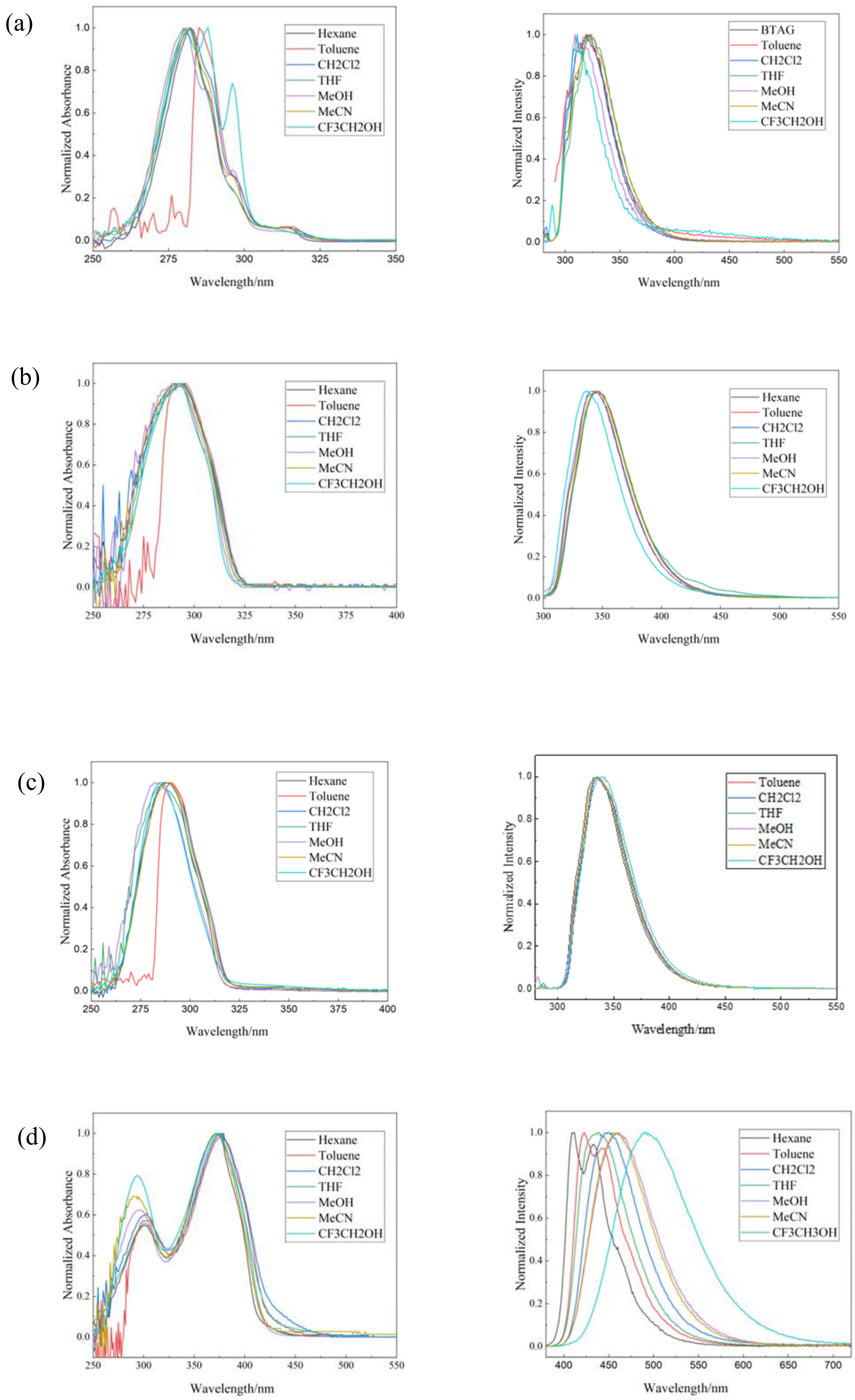

Figure S1. UV absorption (left) and PL spectra (right) of BTAG (a), TTAG (b), TTAG-TMS (c), TTAG-TPA (d) in several solvents.

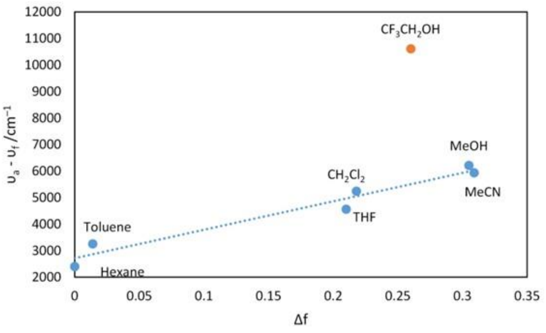

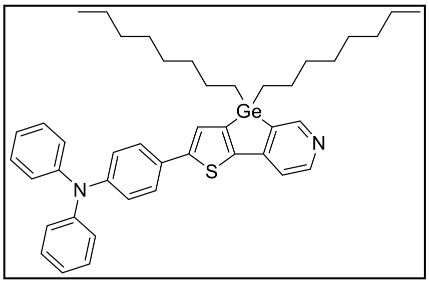

Figure S2. Lippert-Mataga plots (left) and the structure (right) of thienopyridinogermole. The slope for the fit is 10746 cm^{-1} (R^2= 0.96).

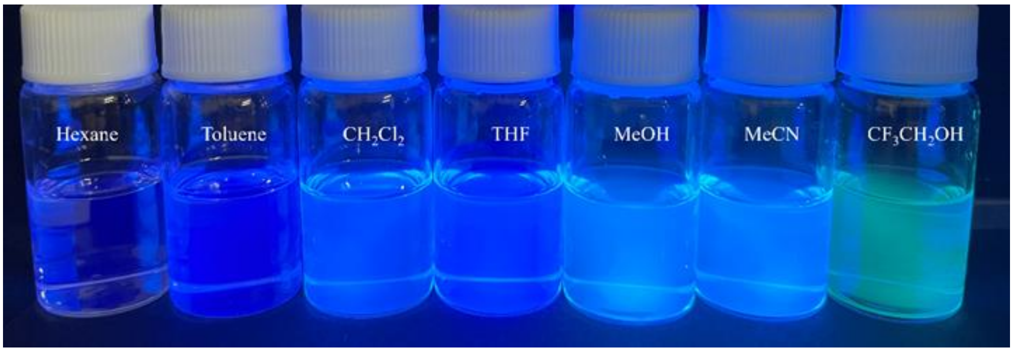

Figure S3. Photo of TTAG-TPA in several solvents under irradiation at 365 nm at room temperature.

Table S1. Absorption maxima, emission maxima and Stokes' shift of TTAG-TPA in various solvents[a]

Solvents	Dielectric constant (ε)	Refractive Index (n)	Δf	λ_a /nm	λ_f /nm	Stokes Shift/cm^{-1}
Hexane	1.88	1.375	0	372	411	2550
Toluene	2.38	1.494	0.014	379	423	2744
CH_2Cl_2	8.93	1.421	0.218	377	449	4253
THF	7.58	1.405	0.210	375	438	3835
MeOH	32.66	1.327	0.309	373	462	5164
MeCN	35.94	1.342	0.305	373	456	4879
TFE[a]	8.55	1.300	0.260	373	492	6484

[a] TFE (trifluoroethanol).

The Lippert-Mataga equation [(S1), (S2)]

$$v_a - v_f = \frac{2(\mu_e - \mu_g)^2}{hc\alpha^3}\Delta f + \text{const} \qquad (S1)$$

$$\Delta f = \left[\frac{\varepsilon - 1}{2\varepsilon + 1}\right] - \left[\frac{n^2 - 1}{2n^2 + 1}\right] \qquad (S2)$$

where ν_a and ν_f are the absorption and fluorescence band maximum positions (cm^{-1}), respectively, ε is the dielectric constant of the solvent medium, and n is the refractive index of the medium. The quantities h, c, a, μg and μe are Planck's constant, the velocity of light, the Onsager cavity radius, the ground and excited state dipole moments, respectively. The detailed parameters of the solvents were taken from references 3 and 4.

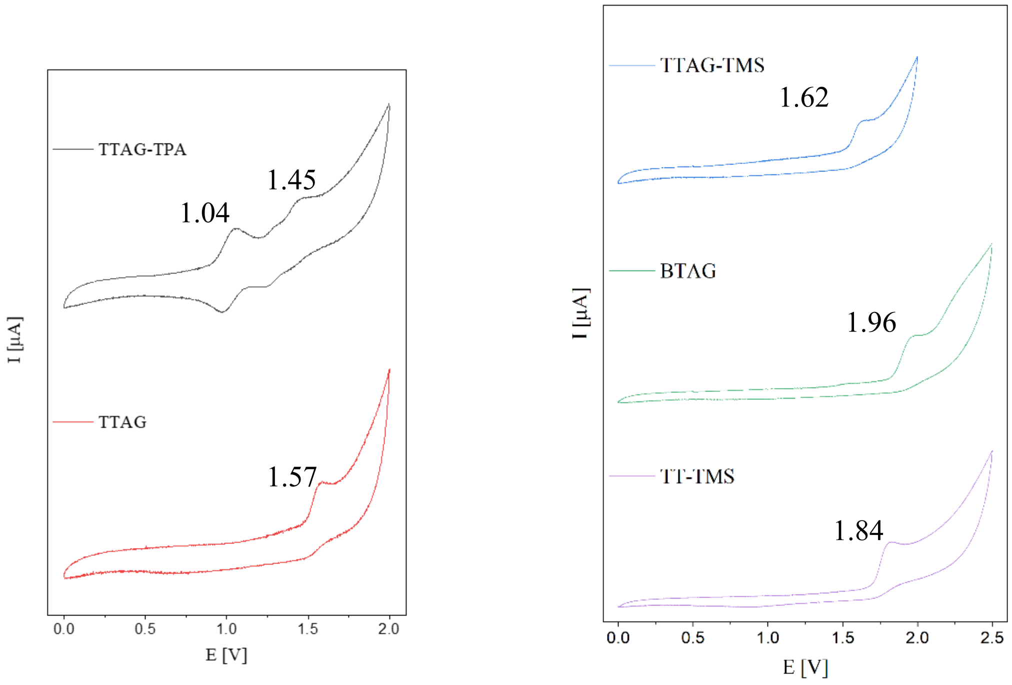

Figure S4. Cyclic voltammograms of BTAG, TTAG, TTAG-TMS, TTAG-TPA and TT-TMS in MeCN/TBAHFP (0.1 M), [c] = $1*10^{-4}$mol L^{-1}, 298K, scan rate = 50 mV s^{-1}.

NMR spectra

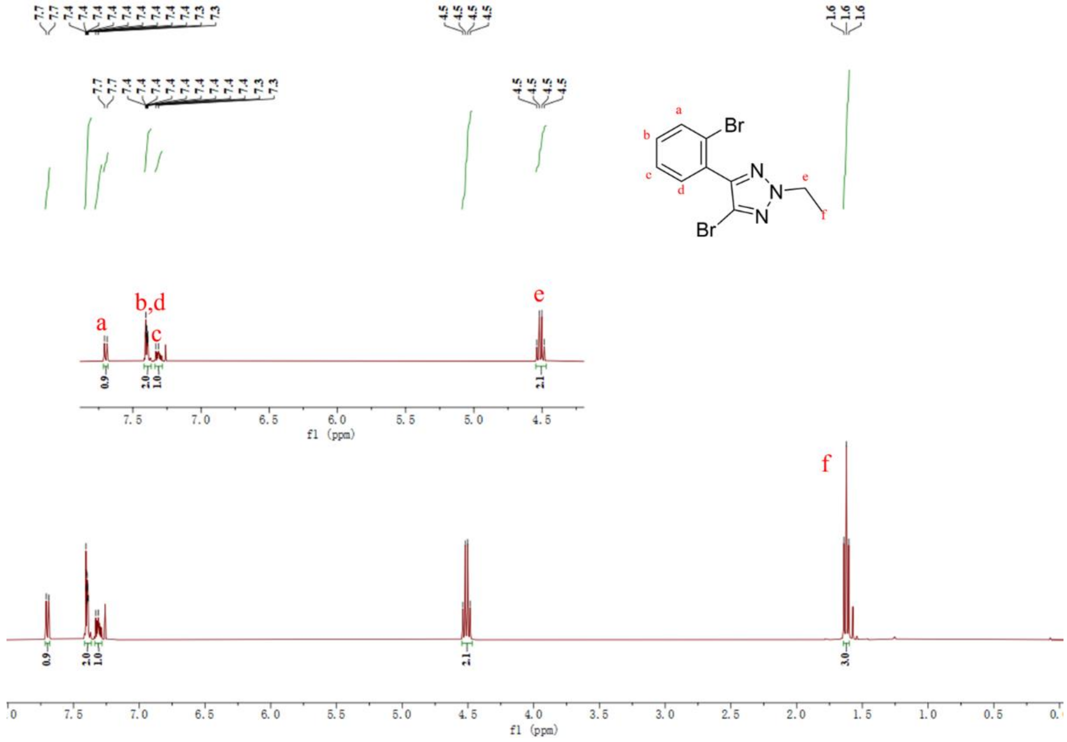

Figure S5. ^{1}H NMR spectrum of **2** in $CDCl_3$.

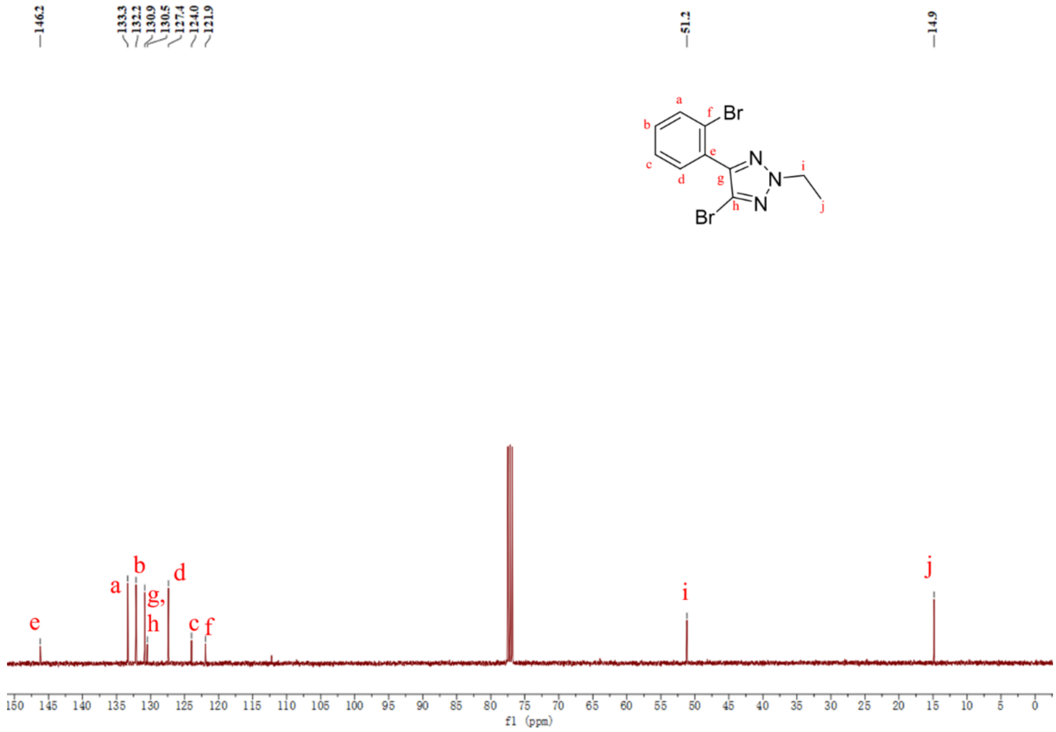

Figure S6. ^{13}C NMR spectrum of **2** in $CDCl_3$.

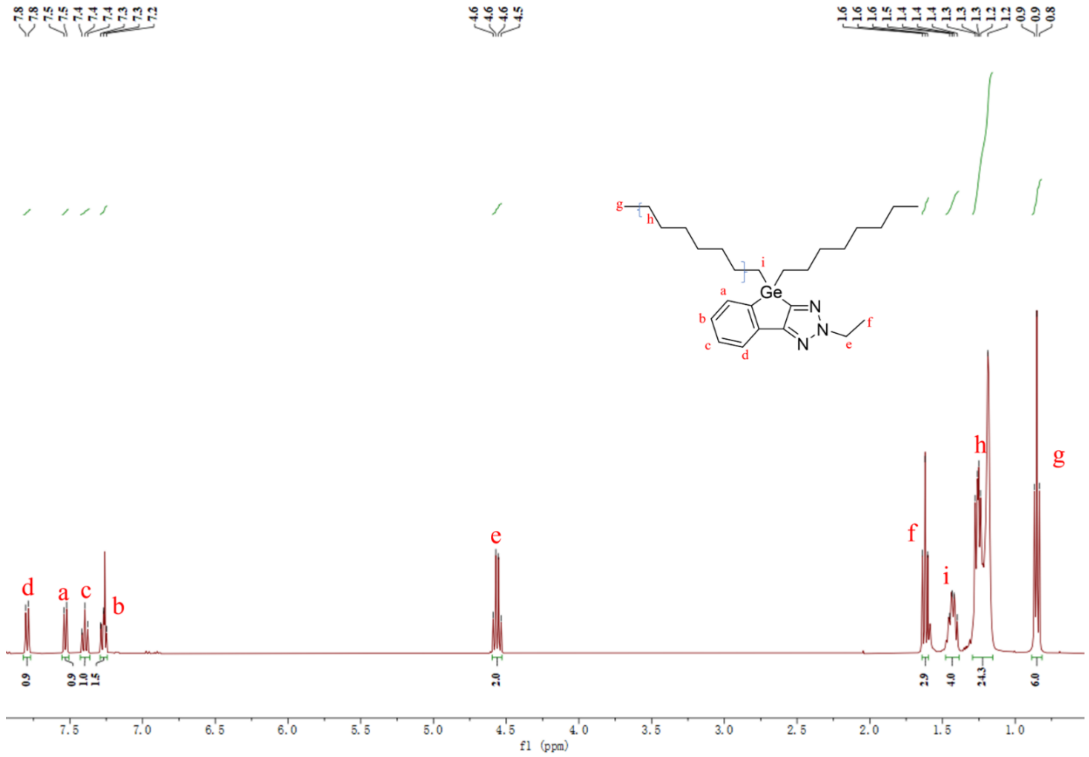

Figure S7. ^{1}H NMR spectrum of BTAG in $CDCl_3$.

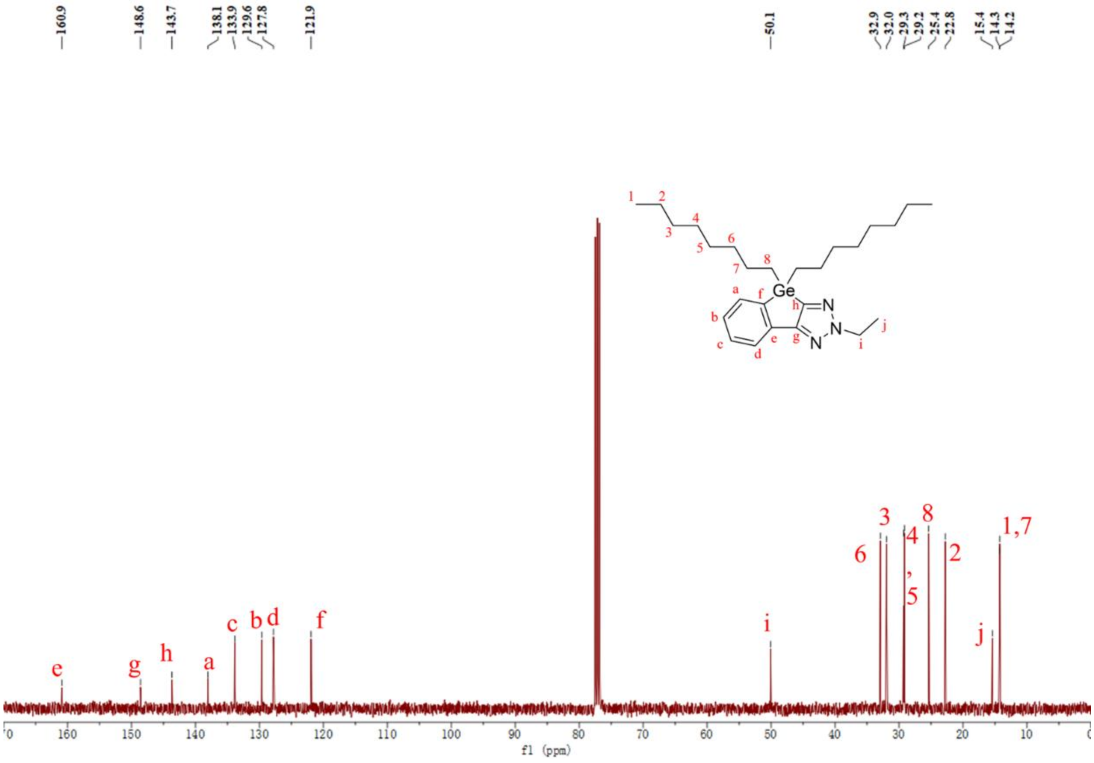

Figure S8. ^{13}C NMR spectrum of BTAG in $CDCl_3$.

6

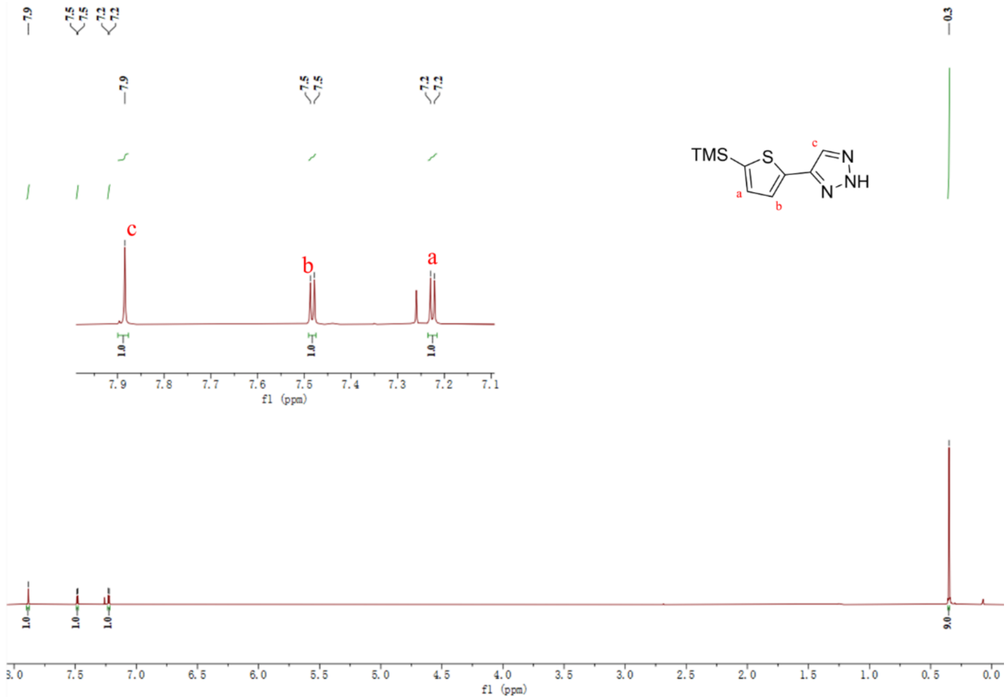

Figure S9. ^{1}H NMR spectrum of **4** in $CDCl_3$.

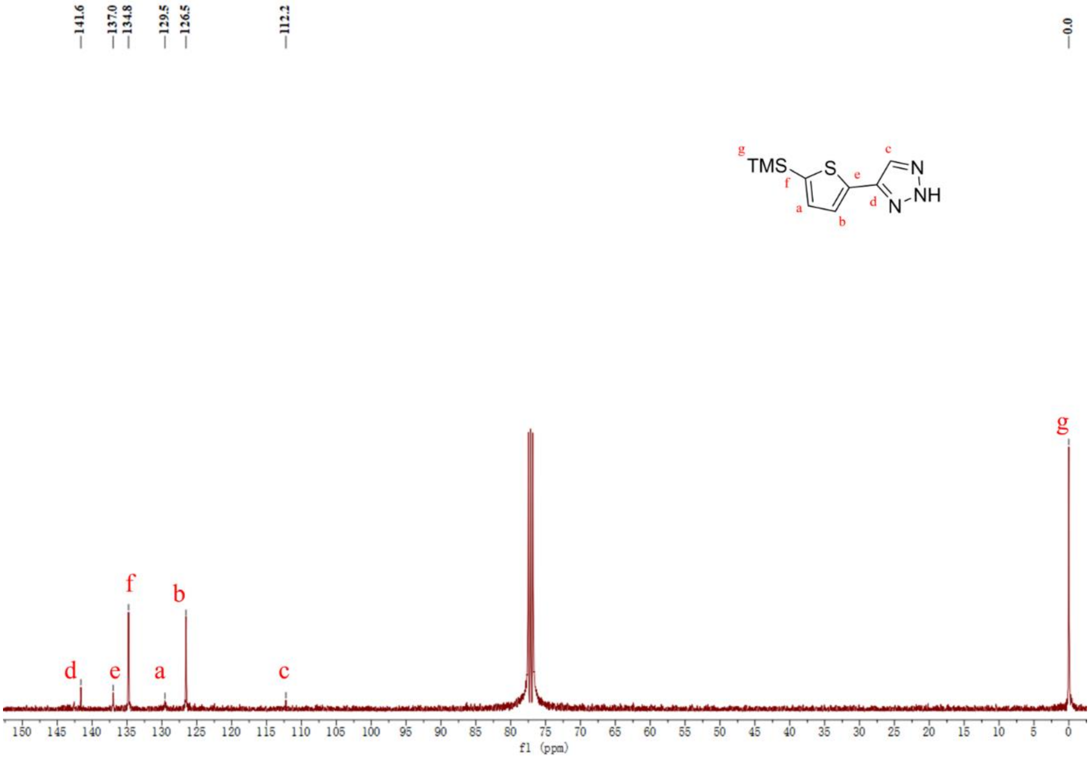

Figure S10. ^{13}C NMR spectrum of **4** in $CDCl_3$.

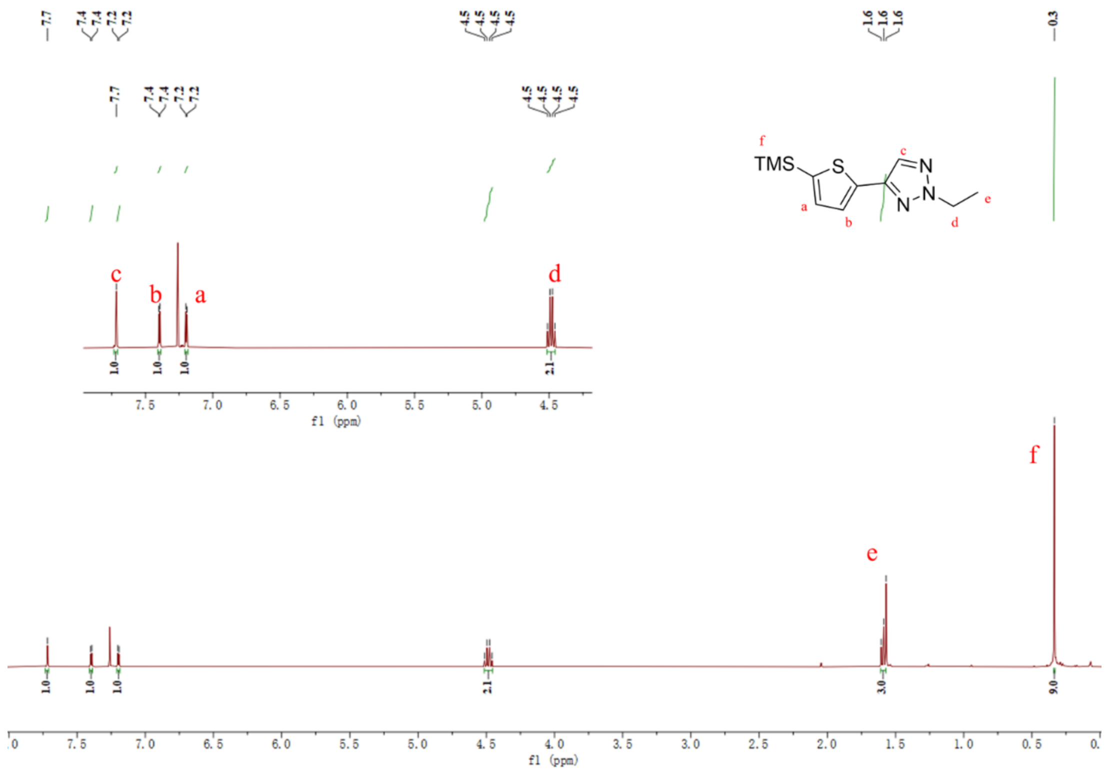

Figure S11. ^{1}H NMR spectrum of TT-TMS in $CDCl_3$.

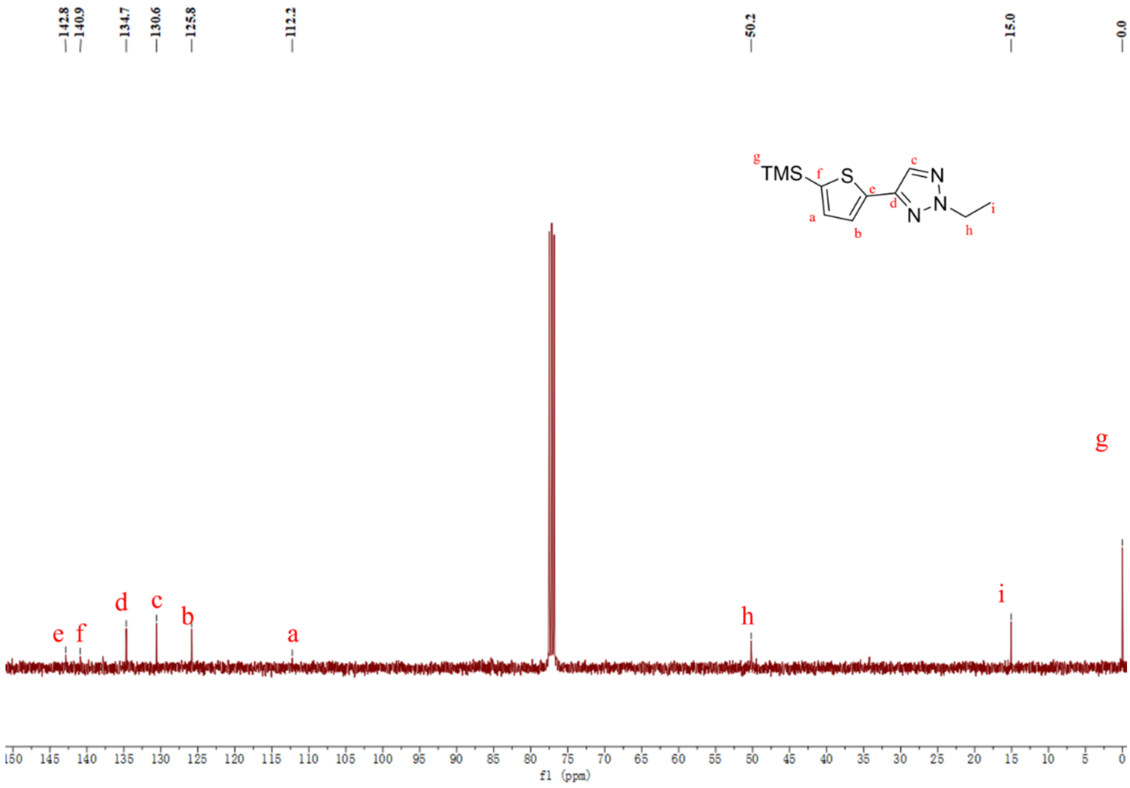

Figure S12. ^{13}C NMR spectrum of TT-TMS in $CDCl_3$.

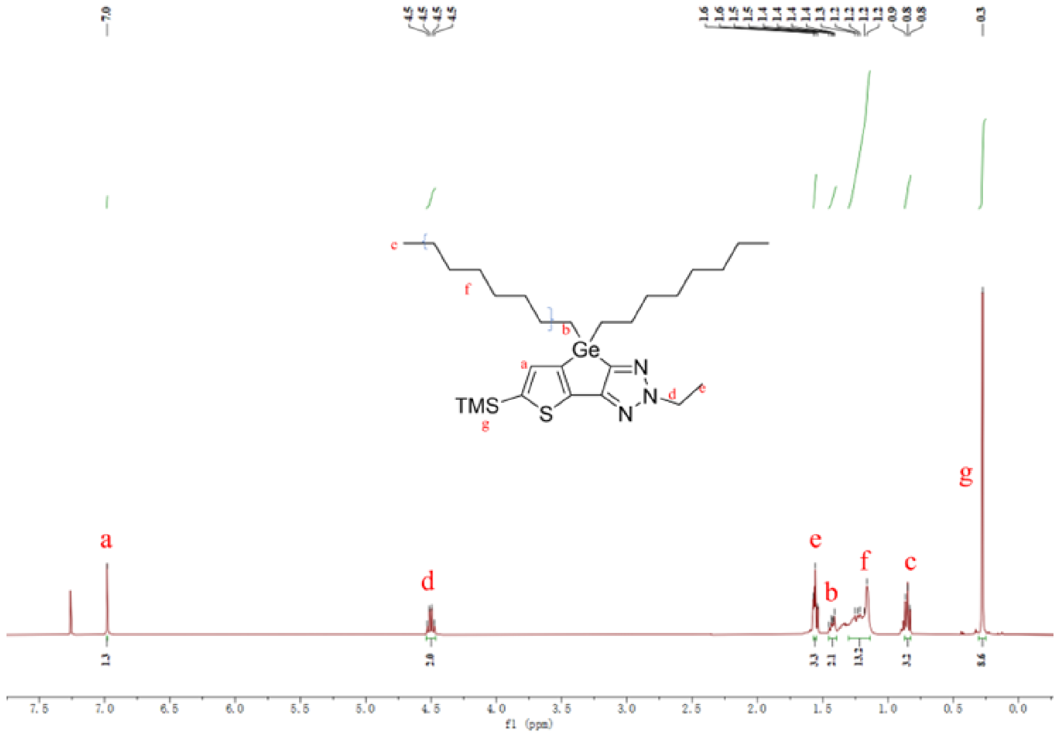

Figure S13. ^{1}H NMR spectrum of TTAG-TMS in $CDCl_3$.

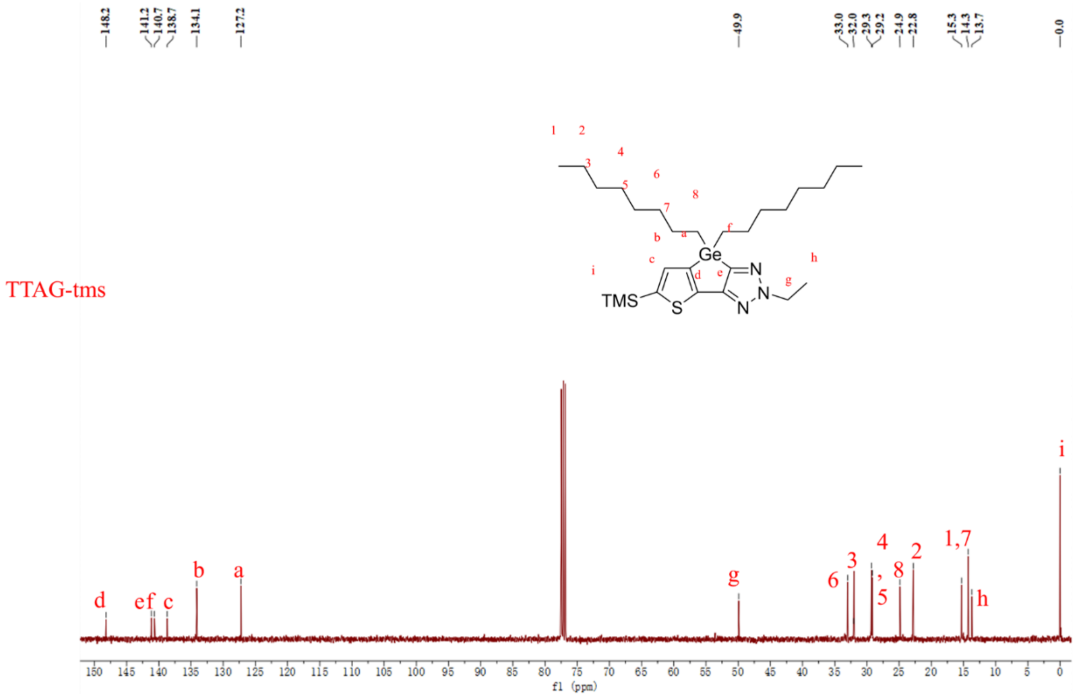

Figure S14. ^{13}C NMR spectrum of TTAG-TMS in $CDCl_3$.

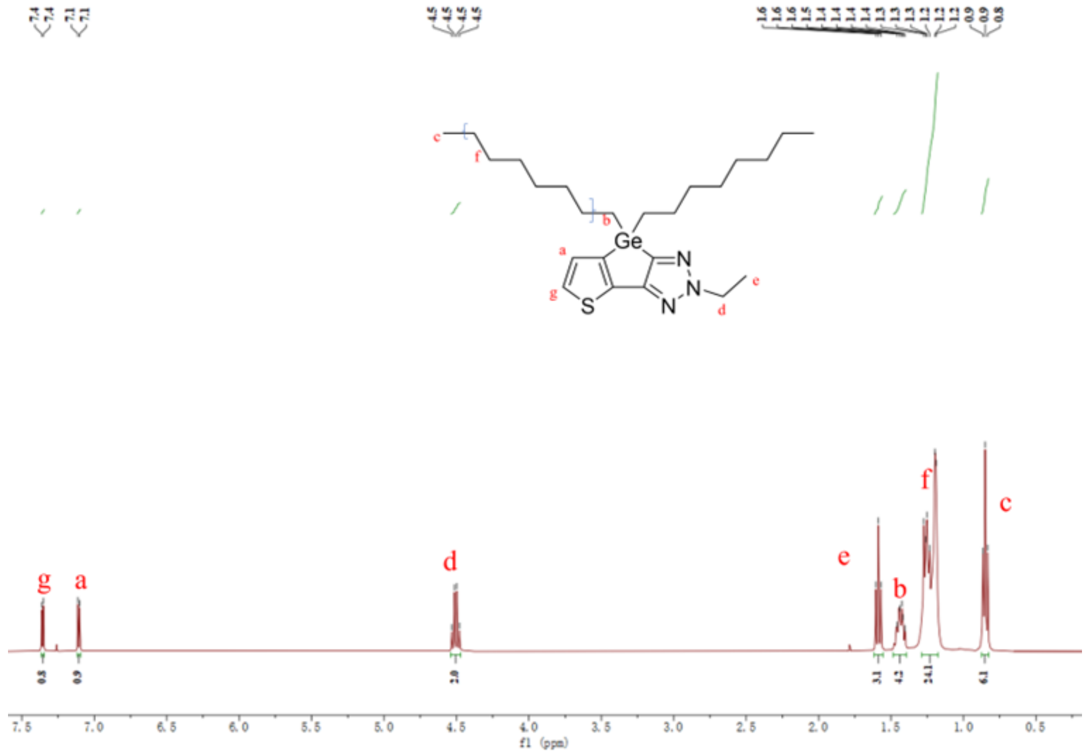

Figure S15. ^{1}H NMR spectrum of TTAG in $CDCl_3$.

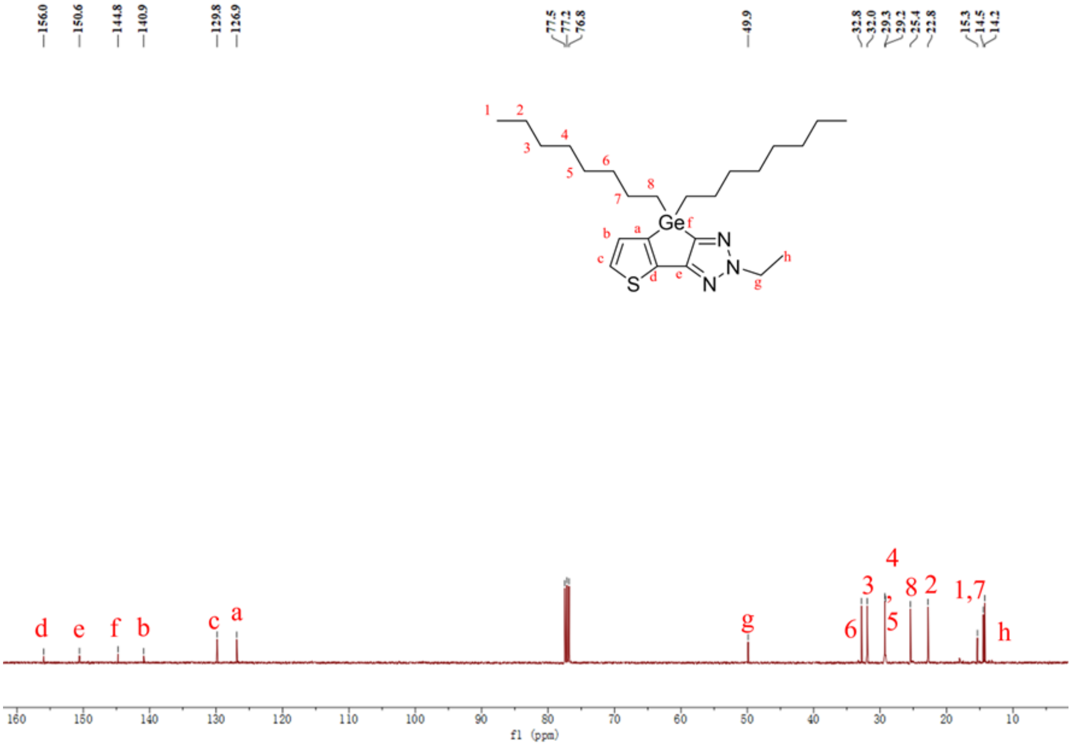

Figure S16. ^{13}C NMR spectrum of TTAG in $CDCl_3$.

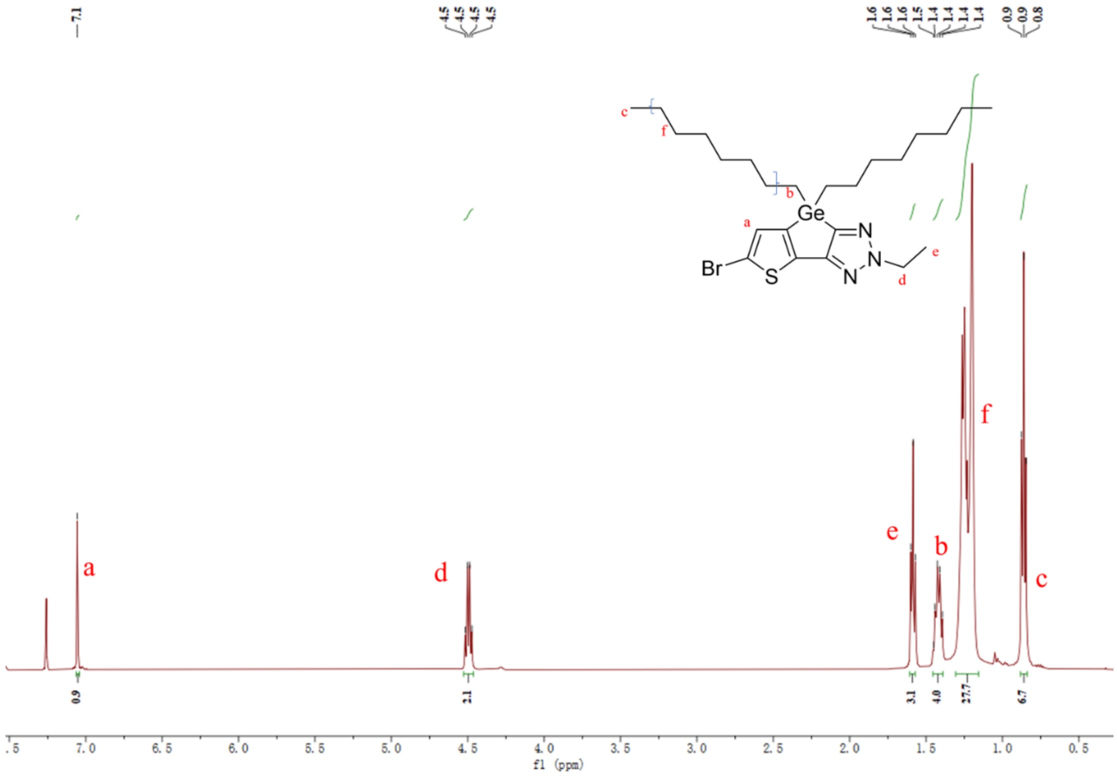

Figure S17. ^{1}H NMR spectrum of **TTAG-Br** in $CDCl_3$

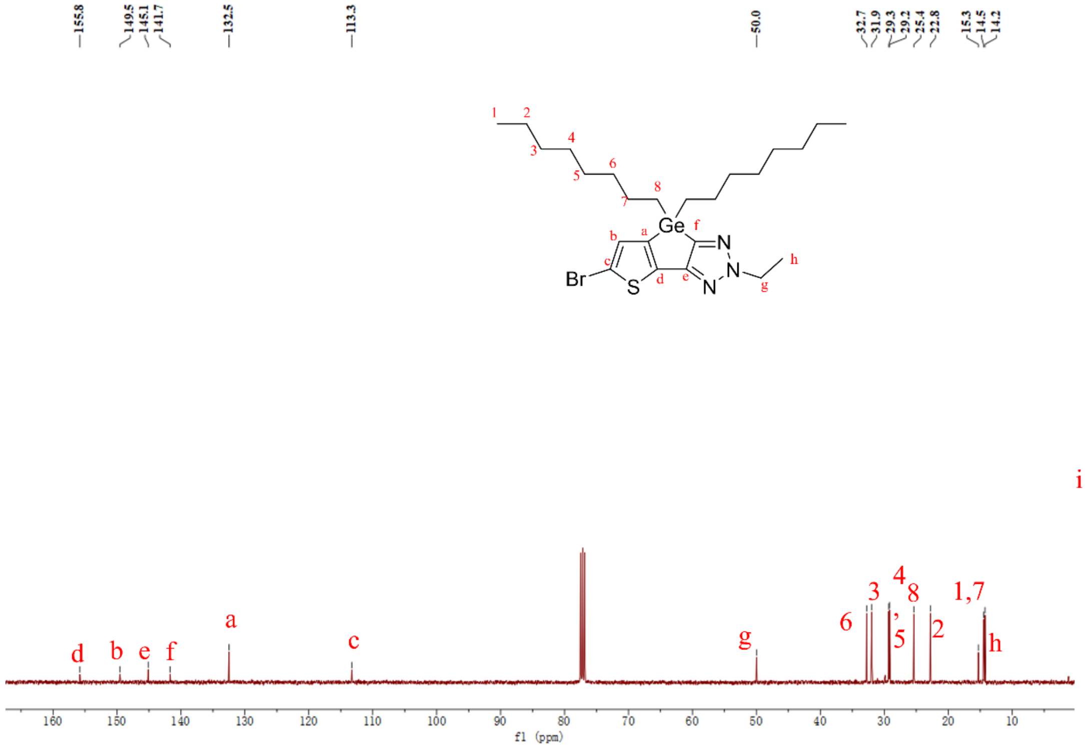

Figure S18. ^{13}C NMR spectrum of TTAG-Br in $CDCl_3$.

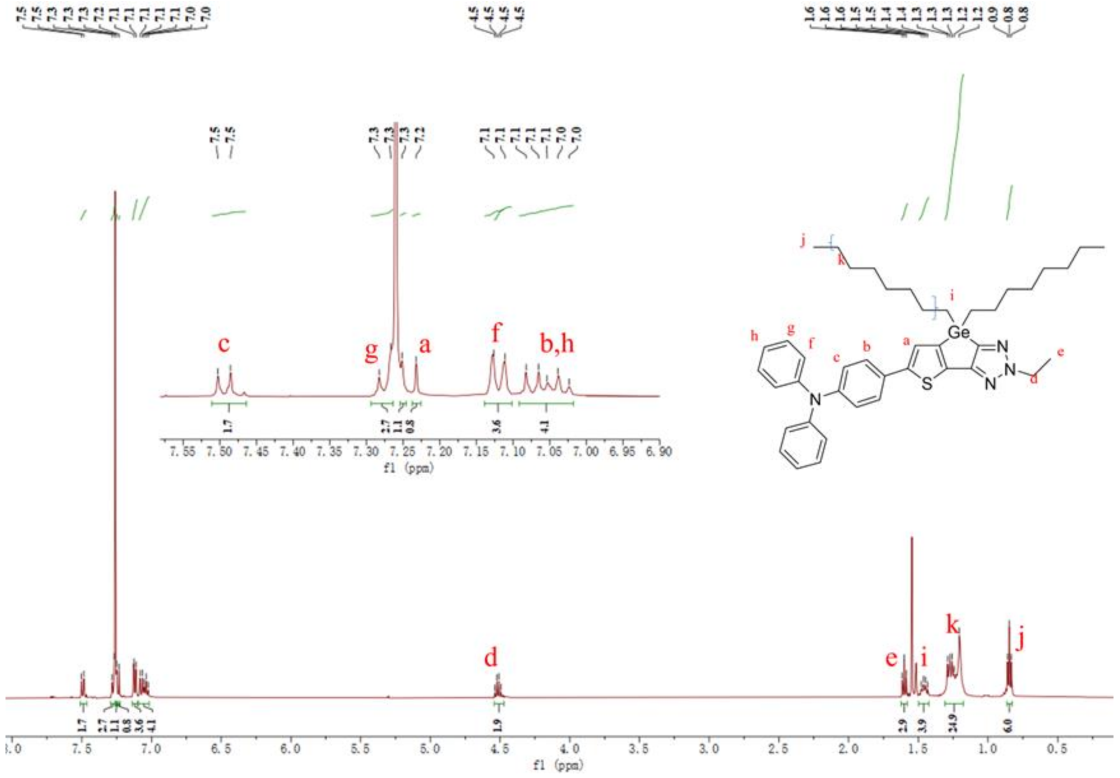

Figure S19. ^{1}H NMR spectrum of TTAG-TPA in $CDCl_3$

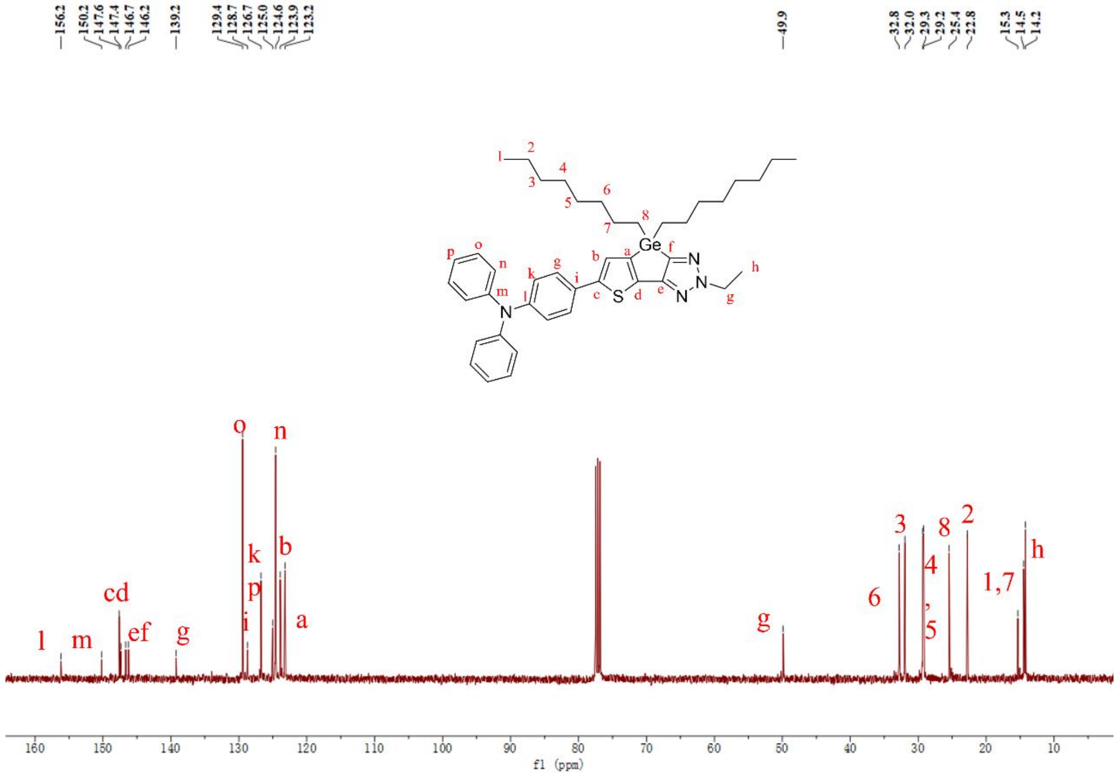

Figure S20. ^{13}C NMR spectrum of TTAG-TPA in $CDCl_3$.

References

1. Hu, Q.; Liu, Y.; Deng, X.; Li, Y.; Chen, Y. Aluminium(III) Chloride-Catalyzed Three-Component Condensation of Aromatic Aldehydes, Nitroalkanes and Sodium Azide for the Synthesis of 4-Aryl-*NH*-1,2,3-triazoles. *Adv. Synth. Catal.* **2016**, *358*. 1689-1693.
2. Denat, F.; Gaspard-Iloughmane, H.; Dubac, J. An Easy One-Pot Synthesis of Group 14 C-Metallated 2 (or 3)-Furan- and Thiophenecarbaldehydes. S*ynthesis*. **1992**, *1992*, 954 - 956.
3. Lide, DR. CRC Handbook of Chemistry and Physics, 87th ed Editor-in-Chief: David R. Lide (National Institute of Standards and Technology). *J Am Chem Soc*. **2006**, *129*, 724.
4. Pandey, N.; Tewari, N.; Pant, S.; Mehata, M.S. Solvatochromism and estimation of ground and excited state dipole moments of 6-aminoquinoline*. Spectrochim Acta A Mol Biomol Spectrosc* **2022**. *267*,120498.
